# Targeting of soluble guanylyl cyclase limits infarct size in a model of acute myocardial infarction

**DOI:** 10.1186/1471-2210-11-S1-P8

**Published:** 2011-08-01

**Authors:** Justin S Bice, Gary F Baxter

**Affiliations:** 1 Division of Pharmacology, Welsh School of Pharmacy, Cardiff University, King Edward VII Avenue, Cardiff, CF10 3NB, UK

## Background

Reperfusion of the myocardium is imperative to limit the damage caused, by occlusion of an artery following myocardial infarction (MI). However, restoration of blood flow to the ischaemic tissue causes damage itself, a phenomenon known as reperfusion injury. Modifying cellular processes at early reperfusion can reduce the irreversible cell damage caused. The reperfusion injury salvage kinase (RISK) pathway, an endogenous signalling pathway that is activated during early reperfusion to limit lethal damage is a pharmacological target. Soluble guanylyl cyclase (sGC) – cyclic guanosine monophosphate (cGMP) is a component of the RISK pathway. Previous studies have shown that the cGMP analogue 8Br-cGMP limits infarct size when given at reperfusion in an experimental model of MI [1]. We therefore hypothesised that administration of the sGC stimulator BAY 41­2272 or the sGC activator BAY 60­2770 at reperfusion would be cardioprotective, limiting infarct size.

## Materials and methods

Hearts from male Sprague-Dawley rats (300-350g) were Langendorff perfused with modified Krebs-Henseleit buffer at constant pressure (74mmHg) [2]. After a period of stabilisation hearts were subjected to 35min regional ischaemia by temporarily occluding the left descending coronary artery. At 30 min ischaemia, BAY 41­2272 or BAY 60­2770 were perfused for 15 min (10 min into reperfusion). Control hearts were perfused with the same buffer in the absence of the drug. To further characterise the infarct limiting properties of BAY 41­2272 and BAY 60­2770, further Langendorff experiments were carried out in which the sGC inhibitor ODQ was perfused with or without BAY 41­2272 and ODQ with or without BAY 60­2770. Following reperfusion hearts were frozen, sliced into disks and stained with triphenyltetrazolium chloride and infarct size was expressed as a percentage of the ischaemic risk zone. To confirm that BAY 41­2272 elevated cGMP, radioimmunoassay was carried out on myocardial tissue samples perfused with BAY 41­2272 at early reperfusion.

## Results

See Figure 1.

## Conclusion

**Figure 1:**
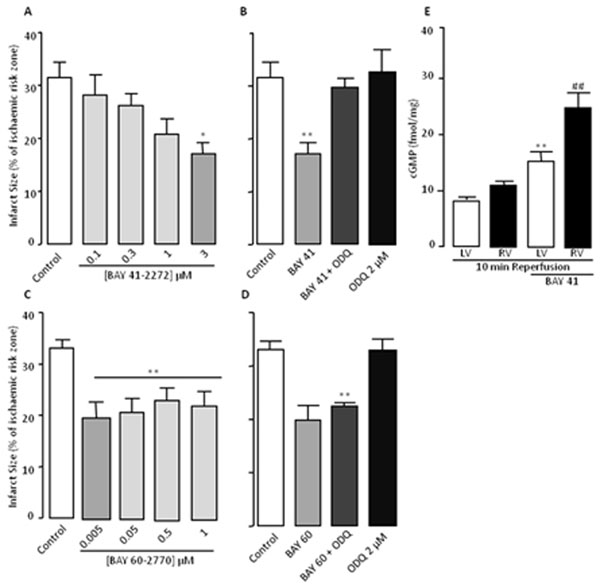
Infarct size data (**A-D**) of hearts perfused with sGC stimulator BAY 41­2272 3 µM unless specified, BAY 60­2770 5 nM unless specified and ODQ 2 µM. cGMP measurements (**E**) of myocardial left ventricular (LV) and right ventricular (RV) tissue samples treated with and with without BAY 41­2272 3 µM at 10 min reperfusion. Data are expressed as mean ± SEM, * p<0.05, vs. Control, ** p<0.01, vs. Control (**A-D**) and ** p<0.01 vs. LV without BAY, ## p< 0.01 vs. RV without BAY (**E**), n≥6 (One-way ANOVA).

The data in Figure 1 shows that both BAY 41­2272 and BAY 60­2770 afford infarct limitation when given at early reperfusion. Protection afforded by BAY 41­2272 requires the presence of the reduced haem moiety, conversely to BAY 60­2770. We also show that BAY 41­2272 elevates cGMP levels in early reperfusion following 35 min left descending coronary artery occlusion. The complex redox balance in early reperfusion following ischaemic insult makes these redox sensitive compounds useful tools when targeting the sGC-cGMP pathway, showing potential for the therapeutic treatment of MI.
